# Stability of Tissue Augmented with Deproteinized Bovine Bone Mineral Particles Associated with Implant Placement in Anterior Maxilla

**DOI:** 10.1155/2019/5431752

**Published:** 2019-10-27

**Authors:** David Buntoro Kamadjaja, Ni Putu Mira Sumarta, Andra Rizqiawan

**Affiliations:** ^1^Department of Oral and Maxillofacial Surgery, Faculty of Dental Medicine, Universitas Airlangga, Surabaya, Indonesia; ^2^Stem Cell Research and Development Center, Universitas Airlangga, Surabaya, Indonesia

## Abstract

**Background:**

Implant placement in defective anterior maxilla poses a great challenge regarding functional and aesthetic outcomes. Therefore, it requires predictable alveolar ridge augmentation. Deproteinized bovine bone mineral (DBBM) particle has commonly been used for bone grafting. However, it is associated with low resorption rates which potentially compromise the outcome of horizontal augmentation in conjunction with implant placement.

**Aims:**

This study is aimed at evaluating the stability of tissue augmented with DBBM particle associated with implant placement in the anterior maxilla.

**Materials and Methods:**

The inclusive criteria consist of patients being treated with guided bone regeneration (GBR) incorporating the use of DBBM particles with either a simultaneous or staged approach. The parameters analyzed include the implant survival rate, post-GBR clinical stability based on tissue resorption level, and the tissue stability between simultaneous and staged approaches. Statistical analysis using Mann-Whitney test is performed with significance determined at *p* value < 0.05.

**Results:**

Seventeen patients with 23 implant placements satisfy the criteria for this study. Simultaneous approach is adopted in 18 (78.3%) implants and a staged approach in 5 (21.7%) implants. The implant survival rate is 100%. The evaluation of horizontal tissue stability reveals a low resorption level in 19 (82.6%) implants, while moderate and high resorption levels are found in 3 (13.0%) and 1 (4.3%) implants, respectively. The statistical analysis shows that the simultaneous approach produces significantly (*p* = 0.005) lower resorption level compared to the staged approach.

**Conclusion:**

Horizontal ridge augmentation using DBBM particles associated with implant placement in the anterior maxilla produces good clinical stability. The stability appears to be higher in the simultaneous approach compared to the staged approach.

## 1. Introduction

Nowadays, increasing numbers of patients are seeking dental implant treatment due to its high success rate which poses significant challenges to clinicians dealing with such treatment. One such challenge in implant placement is tissue deficiency in the anterior maxilla leading to functional, structural, and aesthetic compromises which require horizontal ridge augmentation [1, 2].

Guided bone regeneration technique has been widely employed in horizontal augmentation [3] for which autogenous bone graft has represented the gold standard graft material. However, autogenous bone graft has been associated with limited donor sources and donor site morbidity [4]. Therefore, bone substitutes are now preferred since they permit minimally invasive surgery.

Deproteinized bovine bone mineral (DBBM) particle is a natural bone substitute with good osteoconductive properties which has been shown to achieve impressive results in various experimental and clinical studies [5–8]. However, DBBM has also been associated with very low degradation rates with the result that defect healing is characterized by graft particles being integrated within the new bone, rather than by complete replacement with new bone or bone regeneration [9]. This clinical study attempts to evaluate the stability of tissue augmented with DBBM particle in conjunction with implant placement in the anterior maxilla.

## 2. Subjects and Methods

**Figure 1:**
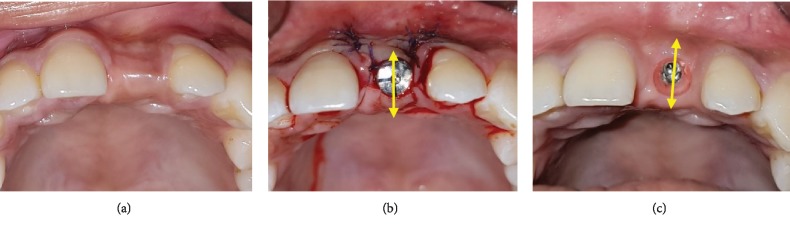
The measurement of augmented tissue resorption. (a) Postextraction horizontal defect of 21 alveolar ridge prior to augmentation; the tissue resorption is established by measuring clinically the difference in alveolar width between the immediate post augmentation procedure (b) and the follow-up at the end of the study period (c). The data collected is subsequently categorized into tissue resorption level as *low* if horizontal loss < 1 mm, *moderate* = 1-2 mm, and *high* > 2 mm.

This retrospective study evaluates patients treated with bone grafting procedures in conjunction with implant placement at the Outpatient Clinic, Dental Hospital, Universitas Airlangga, Surabaya, between July 2010 and August 2018. The study is certified to be ethically cleared by Health Research Ethical Clearance, Faculty of Dental Medicine, Universitas Airlangga (number 288/HRECC. FODM/VIII/2018). The inclusion criteria for the study are as follows. Patients present with reduced horizontal dimension of the alveolar ridge after extraction of the anterior maxillary teeth requiring implant replacement (Figure 1(a)). The augmentation procedure for alveolar defects consists of guided bone regeneration using DBBM particles and resorbable membrane through either a simultaneous approach in which the bone grafting procedure and implant placements are performed at the same time or a staged approach in which the bone grafting procedure is conducted prior to implant placement. Excluded from this study are patients with poorly controlled diabetes mellitus who fail to return for review or whose cases involve the application of bone graft other than DBBM. Informed consent forms are signed by all patients regarding the use of bone substitutes and implant placement.

Data collected during this study includes the distribution of sample sex and age, the duration of treatment in the simultaneous and staged approaches, the length of observation, and the distribution of the number of implant placement related with augmentation approaches. The parameters analyzed comprise the implant survival rate and the stability of alveolar tissue width post augmentation procedure involving the use of resorption level. The tissue resorption is established by measuring clinically the difference in alveolar width between the immediate post augmentation procedure (Figure 1(b)) and the follow-up at the end of the study period (Figure 1(c)). The data collected is then categorized into such scores as *low* if the difference is less than 1 mm, *moderate* = 1-2 mm, and *high* = more than 2 mm.

**Figure 2:**
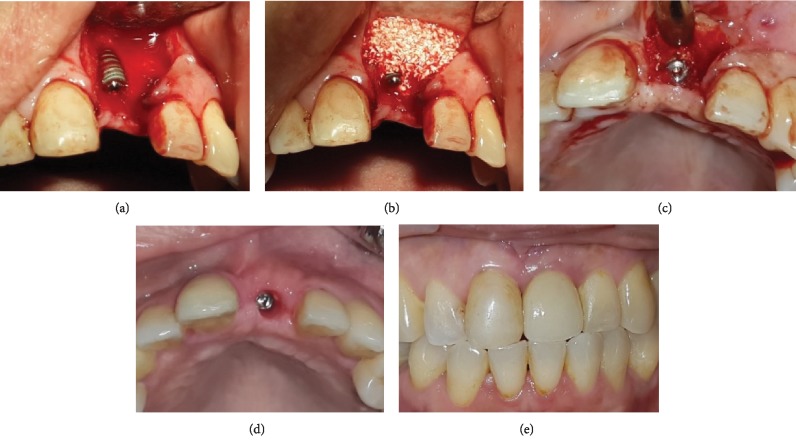
Simultaneous implant placement and horizontal augmentation in the anterior maxilla with delayed loading strategy. (a) Postextraction horizontal defect of 21 alveolar ridge, in which implant placement leaves two-thirds of the implant length exposed; (b) DBBM particle is applied to cover the implant and augment the alveolar defect; (c) at reentry six months after bone grafting, the augmented area is stable with still visible DBBM particles blended with the newly formed bone; (d) at prosthetic phase, the labial tissue width is shown to be comparable to the adjacent tissue; (e) final restoration shows healthy labial and interdental peri-implant tissue.

The surgical procedure within the simultaneous approach uses trapezoidal incision and mucoperiosteal flap in order to expose the defective bone. Bone preparation is performed in a slightly palatal direction as a means of achieving initial implant stability. An osseointegrated implant (Axiom®, Anthogyr, France) is inserted until an initial stability of 35 Ncm torque is achieved leaving implant threads exposed, at least, labially (Figure 2(a)). The peri-implant gap is filled and the horizontal bone defect augmented with DBBM particle (Cerabone®, Botiss GmbH, Germany) (Figure 2(b)). A resorbable membrane (Jason Membrane®, Botiss biomaterials GmbH, Germany) is then placed over the bone graft particle. Healing abutments are installed in the immediate loading, while a cover screw is placed in the delayed loading. Primary closure is facilitated with a periosteal releasing incision. In the staged approach, the bone defects are filled with bone substitute particle, covered with membrane and primarily closed. The graft is allowed to heal for at least 6 months before implant placement is conducted.

In immediate loading cases, implant impression is undertaken approximately 2 months after implant placement. In delayed loading cases, phase 2 is initiated around 6 months post implant placement. Crestal incision is made to expose the implant platform and insert the healing abutment (Figure 2(c)). Approximately one month thereafter, when the soft tissue remodelling around the healing abutment is completed (Figure 2(d)), implant impression is undertaken with closed tray technique for final restoration (Figure 2(e)).

## 3. Results

A total of 17 patients with 23 dental implant placement fulfills the inclusion criteria outlined above, consisting of 9 females and 8 males with a mean age of 43.6 years (range 18 to 66). The length of observation ranged from 8 to 90 months (average 34.9 months). The augmentation was done with simultaneous approach in 18 (78.3%) implant placements and with the staged approach in 5 (21.7%) implant placements (Table 1). Complication documented is wound dehiscence and graft particle exposure in a case treated with a staged approach. A second operation is performed in order to remove the old graft and close the wound without compromising the remainder of the grafted site. The average treatment length in the simultaneous approach was 6.6 months compared to 14.5 months in the staged approach.

The implant survival rate was 100% as no failures are documented during the observation period. Direct horizontal tissue gain is clinically achieved after the augmentation procedure in all cases. The evaluation of clinical alveolar width (horizontal) stability shows low resorption level in 19 (82.6%) implant placements, while moderate and high resorption levels are found in 3 (13.0%) and 1 (4.3%) implant placements, respectively (Table 2).

**Figure 3:**
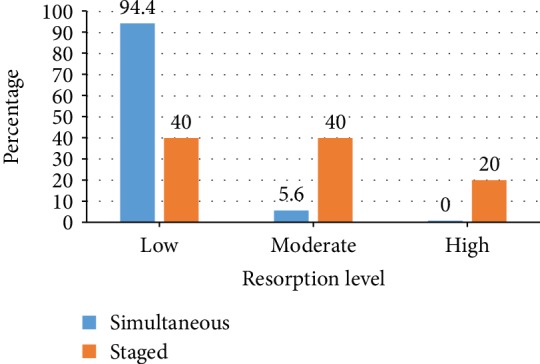
The resorption level of simultaneous and staged augmentation with DBBM particle. The majority (94.4%) of implant augmented with the simultaneous approach shows low resorption level, while only 5.6% is moderate and none is high level. On the other hand, with the staged approach, low resorption level is found in only 40% of implants, while the remainders are moderate (40%) and high (20%) levels. Mann-Whitney test result reveals significant difference (*p* = 0.005) in tissue resorption level between the two approaches.

The evaluation of clinical horizontal tissue stability after GBR procedure using DBBM particle reveals that low resorption level is documented in 94.4% of implant placements augmented with simultaneous approach while moderate resorption level is found in 5.6% implants and none is associated with high resorption level. On the contrary, low and moderate resorption levels are documented both in 40% of implants and 20% of them is related with high resorption level when augmented with the staged approach (Figure 3). Mann-Whitney test result confirms that the resorption level in the simultaneous approach is significantly (*p* = 0.005) lower compared to that in the staged approach.

## 4. Discussion

Implant treatment in the anterior maxilla or aesthetic zone should be performed taking into account one important consideration, i.e., the aesthetic expectations of the patient [10]. One of the major problems associated with early or delayed implant placement in the anterior maxilla is horizontal tissue deficiency due to resorption of alveolar bone especially in the labial aspect. In order to achieve optimal peri-implant tissue support in the anterior maxilla, one should perform bone augmentation with bone graft or bone substitute to provide appropriate stability [11].

The results of this study showed that implant placements grafted with GBR using DBBM produce clinical stability in the horizontal dimension as the majority (82.6%) exhibits low resorption levels during the follow-up review (Table 2). This result is consistent with those of previous studies which reveal good osteoconductivity and stability of the grafted bone [6, 7]. This finding is most likely caused by the high volume stability of DBBM granules which may be associated with its poor degradability. Various studies have demonstrated that DBBM has a very slow degradation rate, up to 3 to 4 years, or may not be completely degradable [12, 13]. Moreover, its stability as graft material can last up to 1.5 years in the absence of dental implants [14].

Alveolar ridge augmentation can be performed at different time points during treatment and is generally categorized as staged or simultaneous approach. The simultaneous approach is obviously the technique preferred by both patients and clinicians since it reduces both the time and cost of treatment [15]. This finding is in accordance with the results of our study in which the treatment duration of the staged approach is more than twice than that of the simultaneous approach.

The result of this study reveals that GBR with the simultaneous approach produces significantly lower resorption level compared to the staged approach. Furthermore, moderate and high resorption levels are predominantly found in the staged approach (Figure 3). This result is noteworthy since DBBM is supposed to be mechanically stable. One plausible explanation would be that the staged augmentation approach is applied to cases with larger tissue deficit which precludes simultaneous implant placement. Larger deficit may have caused a certain degree of instability of the graft particles due to mucosal pressure or mechanical load exerted by provisional prosthesis or mastication [10] resulting in reduced horizontal dimension during the follow-up period. Moreover, the second surgery performed for implant placement in the staged approach might have caused a certain degree of inflammation subsequently inducing bone resorption. These findings highlight the advantages of the simultaneous approach, namely, long-term stability of the grafted tissue compared to that of the staged approach.

The sole use of DBBM to promote augmentation of implant dehiscence might, theoretically, reduce the amount of bone regeneration around the exposed implant surface, thereby potentially compromising implant osseointegration. The DBBM particulate used in this study was made from bovine cancellous bone which demonstrates much higher porosity compared to cortical bone granules. The higher the porosity, the more effective the osteoconductivity of the graft material since it promotes faster bone formation around the exposed implant surface [16]. This is supported by the clinical findings in delayed-loading cases in this study in which good integration between DBBM particle and the new regenerated bone is evident (Figure 2). This suggests that the DBBM particle promotes osteoconduction supporting secondary implant stability during graft healing.

The novel method to measure tissue resorption level employed in this study is a relatively simple means of measuring the stability of grafted tissue. The parameter used in this method is purely based on clinical dimensions not taking into account the magnitude of grafted bone. Furthermore, the sample size used in this study is relatively small and considered heterogeneous to be able to obtain reliably strong inference. Further study is, therefore, required with bigger sample size and with imaging tools enabled to evaluate the magnitude of the grafted bone.

From the results of the study, it may be concluded that horizontal bone augmentation with deproteinized bovine bone mineral particle produces a clinically stable tissue in the anterior maxilla. Stability seems to be higher in the simultaneous approach compared to the staged approach.

## Figures and Tables

**Table 1:** The distribution of implant placement associated with simultaneous and staged augmentation approaches.

	Simultaneous augmentation	Staged augmentation
Male	9	2
Female	9	3
Total	**18** (78.3%)	**5** (21.7%)

**Table 2:** The distribution of tissue resorption level after implant placement associated with simultaneous and staged approaches.

Resorption level	Simultaneous augmentation	Staged augmentation	Total
Low	17	2	**19** (82.6%)
Moderate	1	2	**3** (13.0%)
High	0	1	**1** (4.3%)
Total	**18**	**5**	**23**

## Additional Points

*Key Messages*. Deproteinized bovine cancellous bone mineral particle represents a suitable bone substitute for guided bone regeneration of implant placement in the anterior maxilla due to its strong osteoconductive properties and structural stability. These allow for effective bone integration and stable grafted tissue leading to the effective functioning and aesthetic appeal of implant-supported prosthesis.
